# Comparison of Synthetic Membranes to Heat-Separated Human Epidermis in Skin Permeation Studies In Vitro

**DOI:** 10.3390/pharmaceutics13122106

**Published:** 2021-12-07

**Authors:** Anita Kovács, Stella Zsikó, Fanni Falusi, Erzsébet Csányi, Mária Budai-Szűcs, Ildikó Csóka, Szilvia Berkó

**Affiliations:** Institute of Pharmaceutical Technology and Regulatory Affairs, Faculty of Pharmacy, University of Szeged, Eötvös u. 6, H-6720 Szeged, Hungary; gasparne.kovacs.anita@szte.hu (A.K.); zsikostella@gmail.com (S.Z.); falusi.fanni@szte.hu (F.F.); sooscsanyi@gmail.com (E.C.); budai.szucs.maria@szte.hu (M.B.-S.); csoka.ildiko@szte.hu (I.C.)

**Keywords:** Strat-M membrane, Skin PAMPA, heat-separated human epidermis, Franz diffusion study, skin permeation

## Abstract

In recent years, the study of dermal preparations has received increased attention. There are more and more modern approaches to evaluate transdermal formulations, which are crucial in proving the efficacy of a formulation. The aim of this study was to compare permeation across innovative synthetic membranes (Strat-M and Skin PAMPA membranes) and heat-separated human epidermis (HSE, gold standard membrane) using four different dermal formulations. The Strat-M and Skin PAMPA membranes were designed to mimic the stratum corneum layer of the human epidermis. There have also been some publications on their use in dermal formulation development, but further information is needed. Drug permeation was measured using formulations containing diclofenac sodium (two hydrogels and two creams). The HSE, Strat-M, and Skin PAMPA membranes proved to be significantly different, but based on the results, the Strat-M membrane showed the greatest similarity to HSE. The permeation data of the different formulations across different membranes showed good correlations with formulations similar to these four, which allows the prediction of permeation across HSE using these synthetic membranes. In addition, Strat-M and Skin PAMPA membranes have the potential to select and differentiate a dermal formulation containing diclofenac sodium as an early screening model.

## 1. Introduction

The skin, the largest organ of the human body, provides an easily accessible surface area for the possible administration of drugs, making it an attractive route for both topical and systemic drug delivery. However, dermal drug delivery is a major challenge due to its barrier function [1,2,3].

Numerous guidelines describe the possible test methods for modeling permeation through the skin [4,5,6,7,8]. The most acceptable method for measuring in vitro skin permeation is the use of the test formulation on the surface of the skin model, which is positioned as a barrier between the donor compartment and the receptor compartment of the Franz diffusion cell [9]. The benefits of the in vitro approach are that, in addition to other alternative membranes, measurements can also be carried out on human skin samples. Additional benefits are that several tests can be carried out on a skin sample from the same donor and that many formulations can be tested at the same time.

In dermal permeation tests, human skin is considered a gold standard by regulatory authorities [7]. Despite ethical concerns, human skin is widely used as a model in percutaneous absorption testing. Animal skin models are also utilized as convenient tools to screen an extensive range of drugs, to assess skin permeation-enhancing processes, and to measure the range of skin transport for variety of drug molecules. However, the usage of a broad number of animal and human skin models (e.g., permeation tests in excised animal and human skin) in the scientific literature makes it complicated to appraise the valid difference between the results obtained from various sources. Using biological membranes has further disadvantages, which negatively affect the reliability of formulation screening data. These properties include, for example, variations in skin thickness from skin donors, diseased skin conditions, skin storage circumstances, membrane preparation complexity, hair follicle density, donor age, and high laboratory costs [10,11]. In order to mimic human skin, synthetic artificial membranes are engineered to offer a transparent and reproducible alternative to human and animal skin. Synthetic membranes can easily be designed and stored in contrast to biological membranes. The variability of drug delivery associated with the use of biological skin is also minimized. Owing to their flexibility in deciding thickness, inert nature, composition, ease of handling and storage, and reproducibility in the result of permeation, artificial membranes can substitute human and animal skin models. These advantages, along with ethical concerns, prompted scientists to find alternative ways to minimize the usage of biological membranes in the initial phase of development [12,13,14,15]. There are many synthetic membranes available on the market. Most of these do not provide relevant information compared to measurements on human skin. Typically, these only function as filters with a certain pore size. Thus, in fact, the release of the active ingredient from the formulation can only be measured. However, human skin studies show not only the diffusion profile of the API, but possibly the interaction with the skin and the reservoir function of the stratum corneum as well.

In order for suitable formulations to be placed on the market, studies are needed. The most common measurement method is the use of different diffusion cells. In recent years, there have been increasing efforts to produce membranes with properties similar to human skin. In vitro permeation studies using well-defined skin models and membranes can be useful tools in the design and optimization of skin formulations [12,13,16,17].

The Strat-M membrane has recently become commercially available. It is a special synthetic model for predicting human skin permeation. As a synthetic test model with low variability and no special storage or hydration requirements, the Strat-M membrane simplifies experimental design and data analysis. Like human skin, the Strat-M membrane has multiple layers with different diffuseness, including a very tight surface layer, which is meant to imitate the stratum corneum layer of the human epidermis. The membrane consists of two layers of polyethersulfone (more resistant to diffusion, like the stratum corneum) on top of one layer of polyolefin (more open and diffuse, like deeper layers of the skin). These polymeric layers form a porous structure with a gradient across the membrane in terms of pore size and diffuseness [14,18,19,20,21].

The skin parallel artificial membrane permeability assay (Skin PAMPA) method is a new type of skin permeation test. Skin PAMPA was created to imitate the characteristics of the stratum corneum; for this purpose, the membrane is impregnated with cholesterol, free fatty acid, and ceramide-analogue compounds [1,3,22]. Similarly to the other PAMPA model, this technique is based on a sandwich of two 96-well microtiter plates fitting into each other. The upper plate contains a PVDF filter (~membrane) with a pore size of approximately 45 μm at its bottom [23], which is impregnated with the proper solution of the components mentioned above. The advantage of this model is that it can be considered a low-cost and high-throughput analysis. However, it should be noted that the applicability of the synthetic membrane, in the case of formulation-containing penetration enhancers, needs to be investigated in detail.

The aim of this study was to investigate the synthetic Strat-M and Skin PAMPA membranes and compare them to heat-separated human epidermis. To achieve this, four different formulations (two hydrogels and two creams) were examined. Diclofenac sodium was incorporated in each formulation as API. We wanted to test how well the synthetic membranes would statistically differentiate between various formulations and compare these results to HSE data.

## 2. Materials and Methods

### 2.1. Materials

Polysorbate 60 and propylene glycol were purchased from Sigma-Aldrich (Budapest, Hungary). Methocel E4M (hydroxypropyl methylcellulose) was obtained from Colorcon (Budapest, Hungary). Diclofenac sodium (DFNa), white beeswax, ethanol 96 w/w%, castor oil, cetostearyl alcohol, white petrolatum, liquid paraffin, wool fat, and oleyl oleate were purchased from Hungaropharma Ltd. (Budapest, Hungary). The water used was filtered and deionized water was used (Millipore Milli-Q, Milford, MA, USA).

Excised human skin was collected from a Caucasian female patient who underwent cosmetic abdominal surgery at the Albert Szent-Györgyi Clinical Center (Szeged, Hungary). The technique of in vitro skin permeation does not require ethical approval or the consent of the patient (Act CLIV of 1997 on Health, Section 210/A in Hungary). The Ethical Committee of the University of Szeged was informed of the investigation. The human investigation license code is 83/2008.

### 2.2. Methods

#### 2.2.1. Sample Preparations

Hydrogel No1 (HG1) was formulated with Methocel E4M. It was added to one part of propylene glycol. After waiting for 15 min, water was added to the gel in small amounts under stirring. DFNa was dissolved in the other part of propylene glycol, and this was added to the other part in small amounts under stirring.

Hydrogel No2 (HG2) was prepared in a mixture of purified water and ethanol 96 w/w% with DFNa dissolved. Methocel E4M was added slowly and stirred until gelification.

The oily phase contains cetostearyl alcohol, liquid paraffin, white petrolatum, and polysorbate 60 in the case of oil-in-water cream (O/W). This phase was heated to 60 °C and hot purified water was emulsified into it under stirring. DFNα was added and homogenized until cooled. In the case of water-in-oil cream (W/O), the oily phase contains white beeswax, wool fat, oleyl oleate, and castor oil. After heating (60 °C), purified water was emulsified in the oil phase under stirring. Finally, DFNα was added and homogenized [24].

All formulations contained 1 w/w% of DFNa. The formulations are summarized in the table below (Table 1).

#### 2.2.2. Preparation of Heat-Separated Epidermis

The heat-separation method was applied to isolate the epidermis [25]. The excised human fat-free subcutaneous skin was put in a water bath (60 ± 0.5 °C, 1 min), and the epidermis was separated from the dermis. In order to check the integrity, a visual inspection was performed.

#### 2.2.3. Franz Diffusion Cell Method

The in vitro permeation experiments were performed in a Logan Automated Dry Heat Sampling System (Logan Instruments Corporation, Franklin Township, NJ, USA) with a diffusion region of 1.77 cm^2^ and a receptor medium (PBS pH 7.4) capability of 9 mL. The Franz cell system had thermostatic circulation at a steady temperature of 32 ± 0.5 °C, while the receptor medium was continuously stirred at 500 rpm during the experiment. Strat-M membrane (Strat-M Membrane, Transdermal Diffusion Test Model, 25 mm, Merck KGaA, Darmstadt, Germany) and HSE were used for the permeation tests as membranes. An experiment was conducted for each measured membrane with six diffusion cells. Donor compartments were open during the measurements, which better mimics the conditions of application on real skin. Each membrane was carefully placed at the interface between the donor and the receptor compartments. The amount of the formulation in the donor compartment was about 300 mg. The examination lasted 12 h (sampling times: 0.5; 1; 2; 4; 6; 12 h). The amount of permeated drug was determined at a wavelength of 275 nm with Thermo Scientific Evolution 201 spectrometer using Thermo Insight v1.4.40 software package (Thermo Fisher Science, Waltham, MA, USA) [24]. Measurements were also performed with each drug-free formulation. Data were corrected with the data of drug-free formulations in all cases.

#### 2.2.4. Skin PAMPA Method

The upper skin PAMPA plate (P/N: 120657, Pion, Inc, Woburn, MA, USA), was impregnated with hydration solution (P/N: 120706, Pion, Inc., Billerica, MA, USA) for 24 h before the tests. The donor phase was 70 µL of the preparations and the receptor phase was phosphate buffer solution (PBS pH 7.4 ± 0.10). A total of 250 μL of receptor solution was placed onto the upper plate, and the receptor medium was stirred and incubated at 32 °C during the permeation (Gut-Box™ Pion, Inc., Billerica, MA, USA). The receptor solution was analyzed after 0.5, 1, 2, 4, and 6 h of incubation [26]. The maximum length of the permeation studies was 6 h because the decomposition of the membrane occurred in the case of a longer test time. The permeated DFNa amount was analyzed by means of UV spectroscopy at 275 nm using a SPECTROstarNano UV plate reader from BMG LABTECH GmbH (Ortenberg, Germany).

#### 2.2.5. Permeation Analysis

Permeation profiles were obtained for the four different formulations. The cumulated DFNa quantity (Q, µg) permeated at 6 and 12 h was determined. The slope of the quantities of permeated DFNa (μg/cm^2^) versus time (h) profiles was the flux (J). Timepoint correlations were examined, and correlation coefficients (R^2^) were determined between the quantities of DFNa permeated through the heat-separated human epidermis, the Strat-M, and Skin PAMPA membrane.

#### 2.2.6. Statistical Analysis

Data analysis, statistics, and graphs were performed from the experimental data with Microsoft^®^ Excel^®^ (Microsoft Office Professional Plus 2013, Microsoft Excel 15.0.5023.100, Microsoft Corporation, Washington, USA). Prism for Windows (GraphPad Software Inc., La Jolla, CA, USA) was used to conduct statistical data analysis using the one-way ANOVA variance analysis (Tukey post hoc test). Differences were regarded as significant if * *p* < 0.05, ** *p* < 0.01 and *** *p* < 0.001 versus the control.

## 3. Results

**Figure 1:**
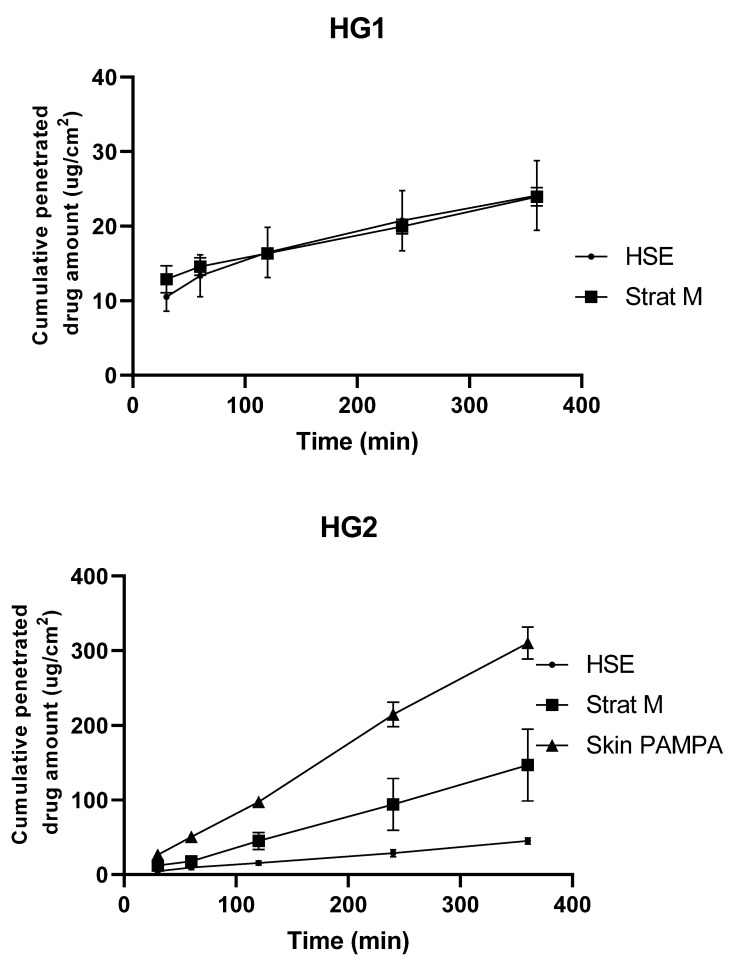

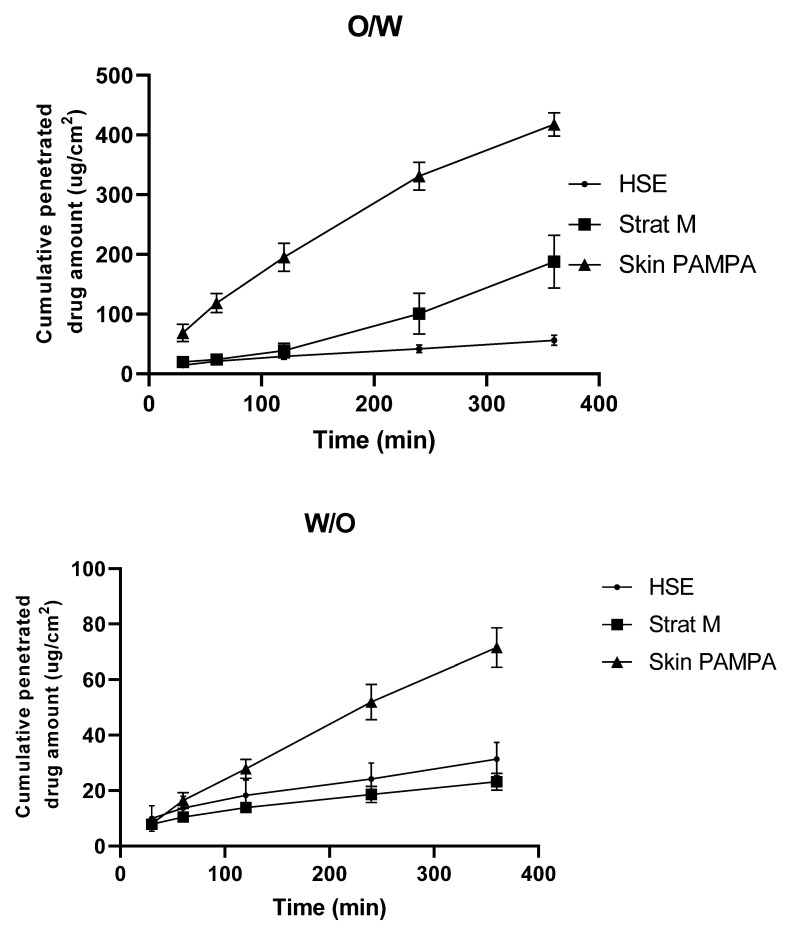
Permeation profile of different formulations using different membranes. Data represent mean and standard deviation.

Permeability is the ability of molecules to permeate through different layers of membranes or skin. In our study, the biological HSE and the new special synthetic Strat-M and Skin PAMPA membranes were compared with each other. The Strat-M, Skin PAMPA membranes and HSE functioned as barriers to API permeation. The permeation of DFNa via the Strat-M, skin PAMPA membranes, and HSE from different formulations is illustrated in Figure 1. It can be clearly seen that the permeation profile of the formulation depends on the applied membrane. In the case of HG1, the results of the Skin PAMPA membrane could not be analyzed because the incompatibility of the membrane and polyethylene glycol was observed during the penetration test. When HG1 and w/o formulations were applied, a slow drug permeation could be seen, and the formulations showed a similar penetration profile through HSE and Strat-M membrane.

When applying alcoholic gel and o/w formulations, fast drug release was observed, where the three different membranes meant different permeation rates during the in vitro test. For both formulations, the fastest permeation was through the Skin PAMPA membrane and the slowest through HSE. In the case of Franz cells, a larger concentration gradient can be expected, as the ratio of donor to acceptor medium is 0.3:10. In contrast, in the case of the PAMPA method, where there is a moderate difference between the two compartment volumes, 70:250, an improved permeation could be observed, which can be explained by a less complex membrane structure.

The linear regression of the permeation curves (Figure 1) resulted in a correlation matrix, presented in Table 2, which indicated similar permeation profiles using different in vitro membranes (Pearson r > 0.95). Based on these, the knowledge of the correlation between membranes allows the interpretation of the assay on synthetic membranes for the assay on HSE.

**Figure 2:**
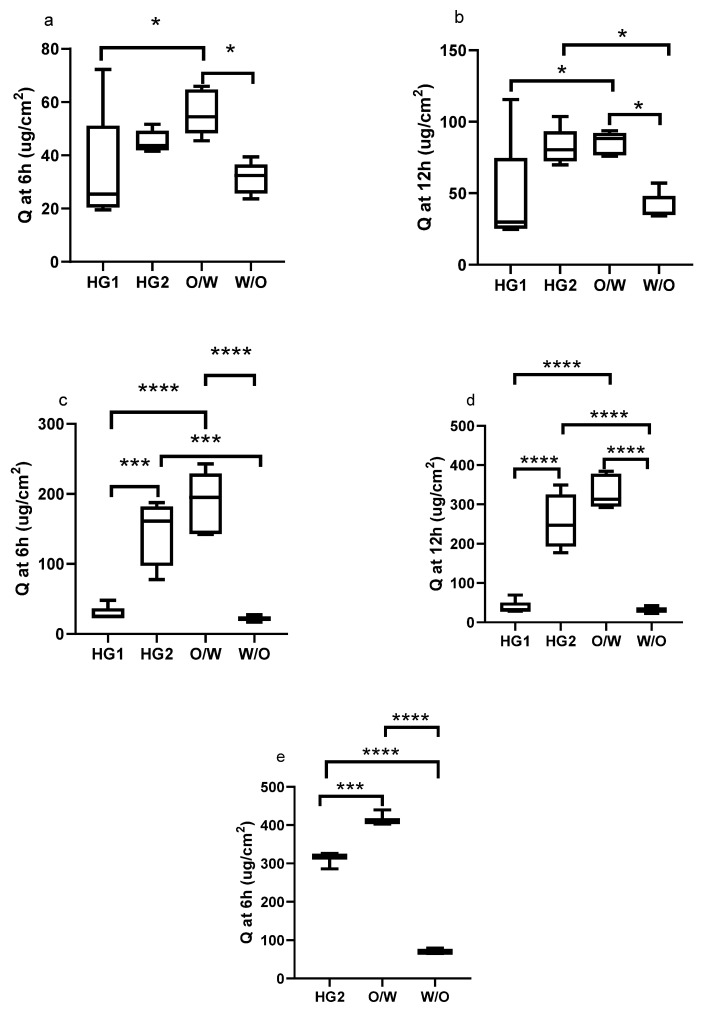
Box diagrams of the permeated drug amount (at 6 and 12 h) from different formulations using HSE (**a**,**b**), Strat-M (**c**,**d**), and Skin PAMPA (**e**) membranes. (* *p* < 0.05, *** *p* < 0.001, **** *p* < 0.0001).

**Figure 3:**
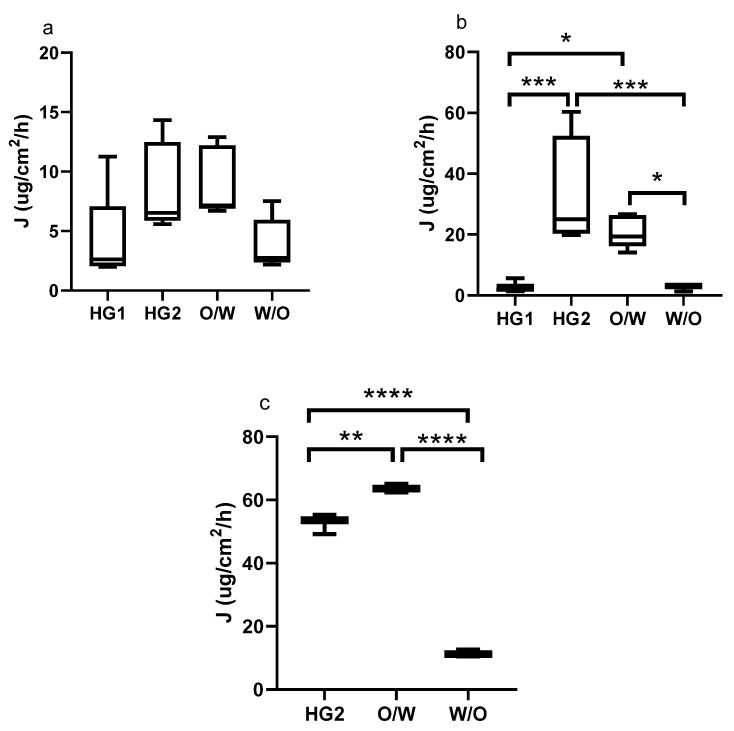
Box diagrams of release rate (Flux, J) from different formulations using HSE (**a**), Strat-M (**b**), and Skin PAMPA (**c**) membranes. (* *p* < 0.05, ** *p* < 0.01, *** *p* < 0.001, **** *p* < 0.0001, ns: no significance).

Permeation values were plotted using a box plot. Box plots present the distribution of numerical data displaying the minimum and maximum data, the data quartiles (or percentiles), and averages. When comparing the formulations, HG2 and o/w cream showed the highest penetrated drug amount (Q), and rate (J) through the membranes in vitro (Figure 2 and Figure 3). These two formulations did not show any significant differences concerning all analyzed permeation parameters (Q at 6 and 12 h, or J). This phenomenon can be explained by different mechanisms, such as (1) the surfactant concentration of the o/w emulsion can facilitate penetration through the membranes; (2) the supersaturation of the evaporation alcoholic hydrogel (HG2) can force penetration; (3) solubility of the API in the formulation; and (4) the complex cream structure can affect the drug diffusion and permeation. The less-active ingredient permeated from the w/o cream and the glycol-containing hydrogel (HG1). These two formulations presented similar permeation parameters; no significant differences could be observed. HG1 contains propylene glycol, which can improve the solubility of DFNa in the donor phase, while the complex cream structure of the w/o cream can hinder the diffusion of the drug. These two phenomena can lead to lower penetration. Donor compartments of the Franz cells were open during the measurements, which better mimics the conditions of application to real skin. The alcoholic gel (HG2) continuously evaporates during the measurements, which leads to an increase in the concentration of the donor phase, so that the evaporation of the propylene glycol-containing gel (HG1) is minimal due to the hygroscopicity of the propylene glycol. An increase in the drug concentration in the donor phase may increase penetration, as indicated by better penetration from the alcohol gel in our results in the case of Franz cell measurements.

When the membranes used are compared in terms of their selectivity to show differences between the different formulations, it can be observed that for Strat-M and Skin PAMPA membranes, a more significant statistical difference can be detected between the compositions, especially in the case of the J value, while applying HSE did not show any significant differences between the four formulations (Figure 3a). This finding suggests that synthetic membranes can differentiate the formulations, but this does not mean it can predict the different penetration across HSE. Due to its natural origin and its complexity, HSE shows low permeability data with high variability, which may mask true differences between the altered formulations.

It should also be noted that the differences between the permeability of HG1 and w/o and between the permeability of HG2 and o/w could not be demonstrated by our permeation studies.

**Figure 4:**
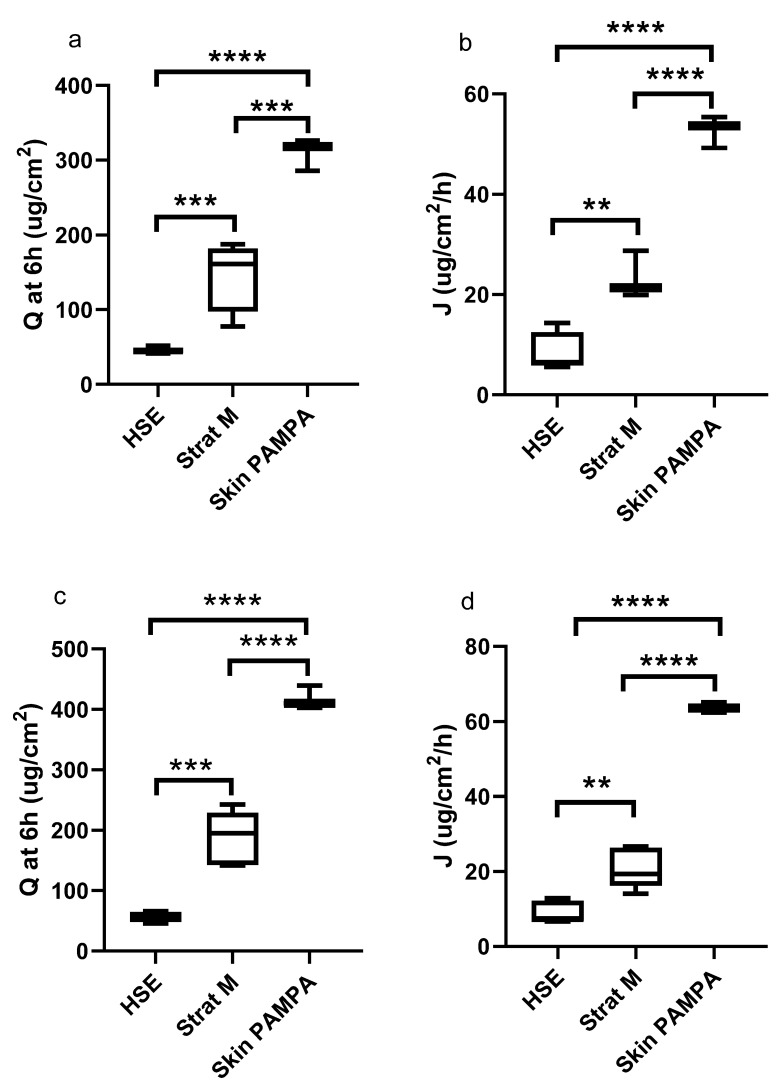

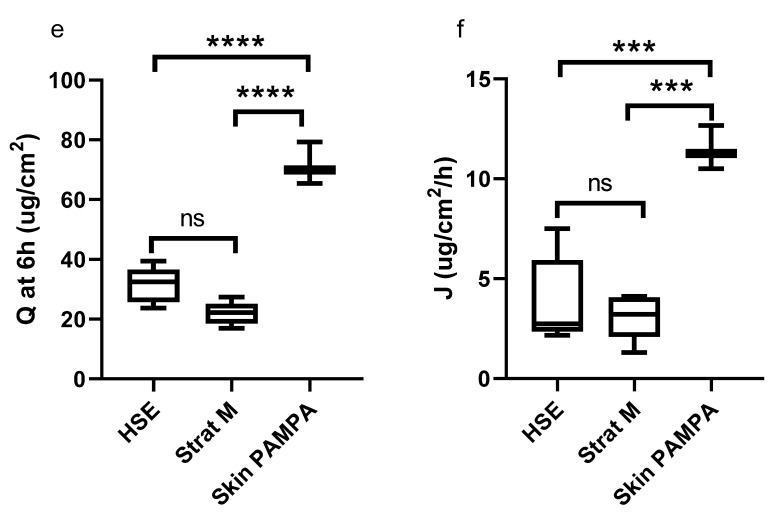
Box plot of the permeation parameters with different membranes in the case of HG2 (**a**,**b**); o/w (**c**,**d**), and w/o (**e**,**f**) formulation. (** *p* < 0.01, *** *p* < 0.001, **** *p* < 0.0001, ns: no significance).

The comparison of the three different membranes revealed that the parameters for the evaluation of drug permeation were significantly different in most cases (Figure 4); only the slow permeation formulation (w/o) presented similar Q and J values (Figure 4b) in the case of HSE and Strat-M membranes.

For all formulations, an increased permeation through the Skin PAMPA membrane was clearly seen, while in general, the amounts permeated through the HSE membrane were the lowest. On comparing the two synthetic membranes, we could detect significantly higher permeation data using the Skin PAMPA membrane.

## 4. Discussion

In our study, Strat-M and Skin PAMPA membranes were applied as synthetic membranes and compared with each other and with HSE.

The Strat-M membrane is composed of multiple layers (stratum corneum, dermis, and subcutaneous tissue mimicking layers) of the membrane, which is similar in structure to human skin. We already have some information about permeation across the Strat-M membrane correlating well with that through human skin [14,15,21] as well as animal skin [27]. In addition, the Strat-M membrane has low batch-to-batch variability, is easy to use, and does not need special storage conditions [28]. In our study, we demonstrated that the permeation profile across the Strat-M membrane was similar to HSE (Table 2), but the general permeation data (Q and J) were significantly higher (*p* < 0.05) (Figure 3) in the case of the Strat-M membrane. The only exception was the w/o formulation, where no significant differences were found. A similar finding was described by Nair et al., namely that the flux value of tocotrienol ethosomes permeation across the Strat-M was significantly higher than that across full-thickness human skin (*p* < 0.05) [29].

The Skin PAMPA membrane is a completely artificial membrane, mimicking permeation through the stratum corneum. It was designed to predict transdermal permeation in a quick, reliable, and cost-effective way [22]. The Skin PAMPA can be used for semisolids [24] and patch formulations [30] as well; it was found to correlate with ex vivo permeation studies [31]. The shortcoming of the Skin PAMPA membrane is that it does not represent the biological complexity of skin, and it does not contain, e.g., proteins, corneocytes, and special lipid subclasses of human skin [32,33]. There are some limitations concerning test length and the applicable material during the PAMPA test because different additives can change the permeability of the membrane. Some information is available about the compatibility of the Skin PAMPA membrane with lipophilic solvents/penetration enhancers, organic acceptor media additives such as isopropyl myristate, dimethyl isosorbide, propylene glycol, diisopropyl adipate, DMSO, and ethanol in the case of the 4 h test length [33], but it is strongly advisable to check the compatibility between the components/media and the membrane at the beginning of the tests. In our case, propylene glycol was applied in HG1 formulation, but during the 6 h test period, presumably due to incompatibility with the membrane, evaluable data were not obtained during the test. Although the purpose of our article was not to investigate possible incompatibilities, it may be important information for comparing the applicability of synthetic membranes. In our work, the permeation profile across Skin PAMPA was similar to that through HSE (Table 2), but the permeation data (Q and J) were significantly different from HSE (Figure 3).

The permeation of different topical formulations (gels and creams) was analyzed across synthetic membranes and HSE; therefore, our statistical analysis allowed the comparison of synthetic membranes with each other and with HSE as well. We found that the permeation profile was similar in each comparison (see correlation matrix in Table 2). Differences can be evaluated in terms of statistical differences. In the case of HSE, in addition to the relatively small permeation, large standard deviations could be observed, which makes it difficult to detect statistically significant differences, so the differences disappear. In contrast, the small standard deviation of the values measured on the synthetic membranes allows a statistical interpretation of the differences. A general order could also be established for permeation data of Q and J: the highest values could be measured for Skin PAMPA, and the lowest values for HSE (Figure 3).

It is important that Strat-M and Skin PAMPA will only provide information on trends and correlations and cannot match absolute permeability values for human skin in every preparation. The permeation efficiency rating helps to identify which methods are the most efficient in developing and optimizing formulations. However, this information can be used to select medications or permeation enhancers or medication/active vehicles in preliminary screening procedures in the pharmaceutical, cosmetic, and personal care industries. Furthermore, skin-mimicking synthetic membranes may be a convenient method for studying and testing the most effective permeation for human skin to be used in vivo. The Strat-M and Skin PAMPA synthetic membranes are quick, cost-effective, easy-to-use, and ready-to-use systems. They can also be used effectively to select the most suitable preparations and to monitor dermal formulations across the human skin as a screening technique. In the pharmaceutical and cosmetic industries, the investigated synthetic membranes could be seen as a cheaper alternative for screening topical formulations and active ingredients. However, their applicability in the case of penetration enhancers needs to be assessed separately.

In this study, the Strat-M membrane seems to have a stronger association in permeation data relative to human skin, indicating its validity as a substitute for human skin, and, in addition, it also makes it possible to present differences in composition during the early development phase.

## 5. Conclusions

In conclusion, this study has shown that Strat-M and Skin PAMPA synthetic membranes have a large correlation with HSE and have the potential to select and differentiate a dermal formulation containing diclofenac sodium as an early screening model. However, it should be noted that when using penetration enhancers, special consideration should be given to evaluating the penetration-enhancing effect during the selection of the appropriate membrane. Although more research is needed on complex topical formulations, these results suggest potential use as an initial screening technique to help the selection of formulations to be studied using a more biologically intact model, thereby helping to develop new topical formulations.

## Figures and Tables

**Table 1:** Compositions of the test preparations.

Hydrogel No1 HG1		Hydrogel No2 HG2		o/w Cream O/W		w/o Cream W/O	
Component	%	Component	%	Component	%	Component	%
Diclofenac sodium	1	Diclofenac sodium	1	Diclofenac sodium	1	Diclofenac sodium	1
Methocel E4M	3	Methocel E4M	3	Cetostearyl alcohol	4	White beeswax	10
Propylene glycol	50	Ethanol 96 w/w%	30	Liquid paraffin	12	Wool fat	10
Purified water	46	Purified water	66	Polysorbate 60	4	Oleyl oleate	5
				White Petrolatum	20	Castor oil	40
				Purified water	59	Purified water	29

**Table 2:** Correlation matrix of the permeation curves of the different formulations applying different membranes.

Pearson r			
HG1			
	HSE	Strat-M	Skin PAMPA
HSE	-	0.9890	-
Strat-M	0.9890	-	-
Skin PAMPA	-	-	-
HG2			
	HSE	Strat-M	Skin PAMPA
HSE	-	0.9970	0.9964
Strat-M	0.9970	-	0.9981
Skin PAMPA	0.9964	0.9981	-
O/W			
	HSE	Strat-M	Skin PAMPA
HSE	-	0.9670	0.9956
Strat-M	0.9670	-	0.9521
Skin PAMPA	0.9956	0.9521	-
W/O			
	HSE	Strat-M	Skin PAMPA
HSE	-	0.9993	0.99583
Strat-M	0.9993	-	0.9967
Skin PAMPA	0.9958	0.9967	-

## Data Availability

The data presented in this study are available in the article.
